# Identification of stable QTLs and candidate genes involved in anaerobic germination tolerance in rice via high-density genetic mapping and RNA-Seq

**DOI:** 10.1186/s12864-019-5741-y

**Published:** 2019-05-09

**Authors:** Jing Yang, Kai Sun, Dongxiu Li, Lixin Luo, Yongzhu Liu, Ming Huang, Guili Yang, Hong Liu, Hui Wang, Zhiqiang Chen, Tao Guo

**Affiliations:** National Engineering Research Center of Plant Space Breeding, South China Agricultural University, Guangzhou, 510642 China

**Keywords:** Direct-seeded rice, Anaerobic germination tolerance, QTL mapping, RNA-Seq, Candidate genes

## Abstract

**Background:**

Anaerobic germination tolerance is an important trait for direct-seeded rice varieties. Understanding the genetic basis of anaerobic germination is a key for breeding direct-seeded rice varieties.

**Results:**

In this study, a recombinant inbred line (RIL) population derived from a cross between YZX and 02428 exhibited obvious coleoptile phenotypic differences. Mapping analysis using a high-density bin map indicated that a total of 25 loci were detected across two cropping seasons, including 10 previously detected loci and a total of 13 stable loci. Analysis of the 13 stable loci demonstrated that the more elite alleles that were pyramided in an individual, the higher the values of these traits were in the two cropping seasons. Furthermore, some anaerobic germination-tolerant recombinant inbred lines, namely G9, G10, G16, and G151, were identified. A total of 84 differentially expressed genes were obtained from the 13 stable loci via genome-wide expression analysis of the two parents at three key periods. Among them, Os06g0110200, Os07g0638300, Os07g0638400, Os09g0532900, Os09g0531701 and Os12g0539751 constitute the best candidates associated with anaerobic germination.

**Conclusions:**

Both the anaerobic germination-tolerant recombinant inbred lines and the loci identified in this study will provide new genetic resources for improving the anaerobic germination tolerance of rice using molecular breeding strategies, as well as will broaden our understanding of the genetic control of germination tolerance under anaerobic conditions.

**Electronic supplementary material:**

The online version of this article (10.1186/s12864-019-5741-y) contains supplementary material, which is available to authorized users.

## Background

Direct-seeded rice (DSR), classified as wet DSR, dry DSR, or water DSR, is becoming increasingly popular across the world due to its cost efficiency and convenience [1]. Compared with wet and dry DSR, water DSR (whereby seeds are broadcasted in standing water) is more advantageous as it is less labor and time intensive, and restrains weed growth. However, rice (*Oryza sativa* L.) is extremely sensitive to anoxia during germination and early growth of the embryo [2–4]. When using the water DSR method, the rice seeds are completely submerged and suffer hypoxia or anoxia, leading to poor or no germination, seedling death, and poor crop establishment [2, 3]. Anaerobic germination (AG) is the main limiting factor for the large-scale adoption of water DSR. Revealing the biochemical and molecular mechanisms of AG tolerance under flooding conditions and breeding rice varieties with superior AG-tolerant traits could effectively resolve the limitations of the water DSR method.

Insufficient energy supply under oxygen-deficient conditions caused by submergence is a major bottleneck for seed germination and seedling survival [5]. α-Amylase is a critical enzyme in the mobilization of starchy reservation in rice. Of the α-amylase members*,* the expression of *RAmy3D* is positively correlated with coleoptile elongation and seedling survival, particularly in tolerant genotypes [3, 6], indicating that *RAmy3D* may be active during the anaerobic mobilization of rice. However, the sequence variations of *RAmy3D* that are associated with the AG tolerance in different rice varieties are still unknown. Moreover, the maintenance of higher α-amylase activity has been widely reported as a significant step in carbohydrate catabolism under submergence [7, 8]. High expression of *CIPK15*, which encodes protein kinases, activates energy and the stressor receptor SnRK1A*,* resulting in a series of downstream cascade reactions, such as the induced expression of the transcription factor MYBS1, thereby enhancing the expression of α-amylases genes [9].

Under hypoxic conditions, ATP is produced through fermentative metabolism using ethanol and available carbohydrates [10]. Two enzymes related to alcohol fermentation metabolism, namely pyruvate decarboxylase (PDC) and alcohol dehydrogenase (ADH), are induced by submergence, and the enzyme activity of tolerant material was found to be higher than intolerant material after 12 h of imbibition [3]. Overexpression of PDC (*pdc1*) in the rice cultivar “Taipei 309” improved anoxia tolerance as a result of an increase in alcohol metabolism [11]. A point mutation in the *Adh1* gene reduces ethanol dehydrogenase activity and leads to a lack of ATP, thereby inhibiting the elongation of the coleoptile sheath of the *reduced adh activity* (*rad*) mutant under submerged condition [12].

Recently, the trehalose-6-phosphate (T6P) phosphatase gene (*OsTPP7*) that is involved in T6P metabolism was cloned [13]. Under anaerobic stress, *OsTPP7* increased the turnover of T6P, thus enhancing starch mobilization and driving the growth kinetics of the germinating embryo and the elongation of the coleoptile, which consequently enhanced AG tolerance. However, the exact mechanism of tolerance to AG is still not fully understood and warrants further investigation [14]. Rice has a very complex molecular regulatory network underpinning AG tolerance and metabolization under anaerobic fermentation. This regulatory network involves numerous factors relating to hemoglobin, expansin, reactive oxygen species, pyrophosphate, nitrite, ferrous ions, nitric oxide, pH reduction (acidification), and lipid peroxidation [15, 16], which are only understood to a limited extent.

To determine the genetic basis of AG in rice, quantitative trait locus (QTL) mapping and genome-wide association study (GWAS) were recently used to identify some QTLs associated with AG. Several studies assigned the elongation of the coleoptile as an indicator trait of the tolerance phenotype in QTL mapping [17–21]. There are other studies assigned the seeding survival rate as an indicator of the tolerance phenotype [4, 22–25]. Some valuable QTLs were obtained by QTL mapping with the two kind of indicator. GWAS is a popular and highly efficient strategy for dissecting complex traits, but few reports regarding the excavation of AG tolerance loci via GWAS are available. A GWAS using 36,901 single nucleotide polymorphisms (SNPs), and identified 88 loci associated with AG tolerance [26]. Through GWAS of 5291 SNPs in 432 *indica* varieties, a total of 15 AG tolerance loci were detected by another report [27].

Although numerous AG tolerance loci have been identified, only one QTL *(qAG-9-2)* has been fine mapped and cloned as *OsTPP*7*.* Therefore, a large gap between the identification of AG tolerance genes and the breeding of DSR rice varieties still exists. This gap is mainly due to the majority of reported loci being identified based on low-density markers, and few reliable and stable AG tolerance loci have been screened for gene cloning. It is thus imperative that ultra-high density markers are used to evaluate stable AG tolerance loci for further investigation.

In this study, a high-density genetic map consisting of 2711 bin markers obtained via the sequencing-based genotyping of 192 recombinant inbred lines (RILs) derived from a cross between the *japonica* cultivar 02428 and *indica* cultivar YZX was used for QTL mapping. The aims of the study were to identify stable QTLs for AG tolerance and screen candidate genes in the early and late cropping seasons (ES and LS). These QTLs will provide a foundation for further investigation of the molecular mechanisms underlying anoxia tolerance and will provide target loci for improving the varieties via molecular breeding. We also performed transcriptome expression profiling and identified differentially expressed genes located in the AG tolerance-related QTL regions, providing valuable information for candidate gene verification and the dissection of gene regulatory networks affecting rice AG tolerance.

## Results

### Phenotypic performance of the parents and RIL population for AG tolerance

**Fig. 1:**
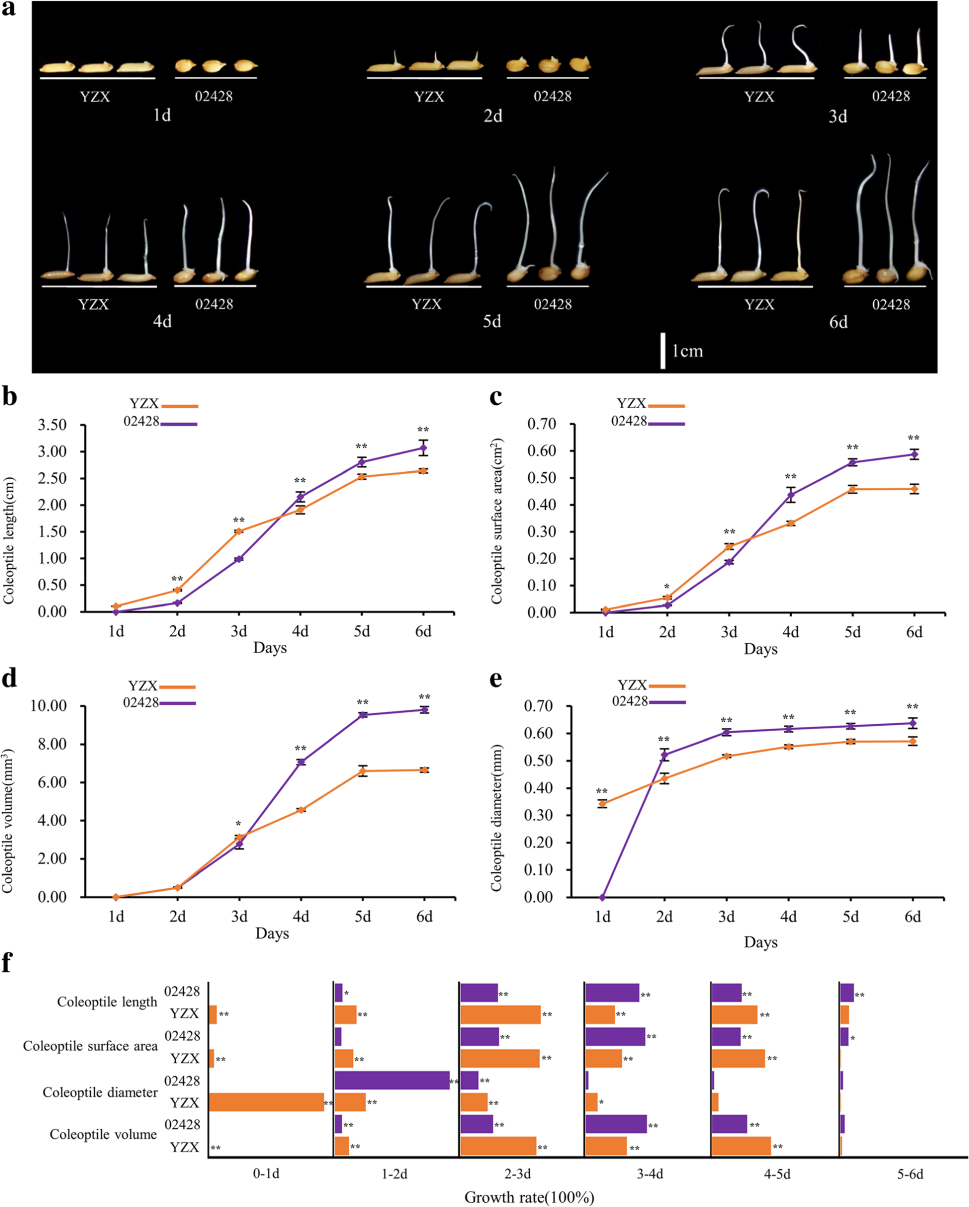
Dynamic phenotypic changes in the coleoptiles of YZX and 02428 under anaerobic conditions. **a**, Phenotype of two parents for 1–6 d under anaerobic conditions; **b**, **c**, **d**, and **e** represent the changes in the length, surface area, diameter, and volume of the coleoptiles in 1–6 d under anaerobic conditions; **f**, The percentage daily increase in the length, surface area, diameter, and volume of the coleoptiles of YZX and 02428 under anaerobic conditions

The faster the rice coleoptile elongates, the sooner the seedling is able to escape the anoxic environment, which improves the chances of survival. Therefore, rice coleoptiles are a classical organ used for studies on AG tolerance [28]. In this study, we investigated the dynamic phenotypic changes in the coleoptiles in two parents under anaerobic conditions at ES. Considerable distinct variations in the coleoptile traits between YZX and 02428 were observed (Fig. 1).

Since YZX germinates faster than 02428, the coleoptile of YZX can be observed on the first day, while the coleoptile of 02428 will be seen on the second day. Although the coleoptile of YZX emerged earlier than that of 02428, the coleoptile length (CL), coleoptile surface area (CSA), coleoptile volume (CV), and coleoptile diameter (CD) of 02428 were significantly greater than that of YZX at the end point (6th day). From the 3rd day, CL, CSA, and CV in 02428 increased significantly faster than in YZX. However, the growth of CD in 02428 exceeded that of YZX from the 2nd day. CL, CSA, and CV growth in both YZX and 02428 occurred mainly from the 2nd to the 5th day. In particular, coleoptile development in YZX mainly occurred from the 2nd to the 3rd day, while coleoptile growth in 02428 was relatively uniform between the 2nd and 5th day. The CD of the two parents differed slightly once the coleoptile had broken through the seed coat (from the 2nd to 6th day) and differed significantly from CL, CSA, and CV.

There were significant or highly significant differences in the four traits between YZX and 02428 at ES and LS (Table 1), indicating large genetic differences between the two parents, which is conducive for QTL mapping. The four coleoptile traits in the RIL population exhibited different degrees of transgressive segregation (Additional file 1: Table S1). Specifically, the variation in CV was the highest of all the traits across the two cropping seasons. The absolute skewness and kurtosis values of each trait in the RIL population were very close to 0, indicating that all of these traits were normally distributed and were controlled by minor multiple genes. In addition, the correlation among the four coleoptile traits in the RIL population was analyzed (Additional file 2: Table S2). With the exception of CL and CD at LS, all the other traits were extremely significantly correlated.

**Table 1:** Phenotypes of YZX and 02428 and the YZX × 02428 RIL population across two cropping seasons

Trait^a^	Environment^b^	Parents^c^		RIL population				
		02428	YZX	Mean	Range	Skewness	Kurtosis	*CV*^d^(%)
CL (cm)	ES	3.07 ± 0.18	2.64 ± 0.05^**^	2.81	1.22–3.88	− 0.32	− 0.38	19.32
	LS	2.59 ± 0.10	2.25 ± 0.17^*^	2.33	0.95–3.76	0.08	0.15	20.77
CSA (cm^2^)	ES	0.59 ± 0.02	0.46 ± 0.02^**^	0.51	0.16–0.81	−0.15	− 0.28	24.37
	LS	0.57 ± 0.02	0.42 ± 0.02^**^	0.44	0.16–0.79	0.22	0.46	22.83
CD (mm)	ES	0.64 ± 0.02	0.57 ± 0.02^**^	0.57	0.45–0.73	0.08	−0.37	9.06
	LS	0.71 ± 0.02	0.59 ± 0.01^**^	0.60	0.49–0.72	0.14	−0.85	8.74
CV (mm^3^)	ES	9.80 ± 0.21	6.65 ± 0.14^**^	7.49	1.95–14.85	0.49	0.07	31.58
	LS	10.19 ± 1.23	6.21 ± 0.21^**^	6.65	2.36–13.30	0.42	0.50	27.45

^a^Trait refers to the names of coleoptile phenotypes under hypoxic conditions: CL coleoptile length, CSA coleoptile surface area, CD coleoptile diameter, CV coleoptile volume

^b^Environment: ES is the early cropping season in 2016; LS is the late cropping season in 2016

^c^Parent refers to the mean ± standard deviation (SD) of the parents, * and ** indicates significance at the levels of 0.05 and 0.01, respectively

^d^*CV:* coefficient of variation

### Identification of QTLs

**Fig. 2:**
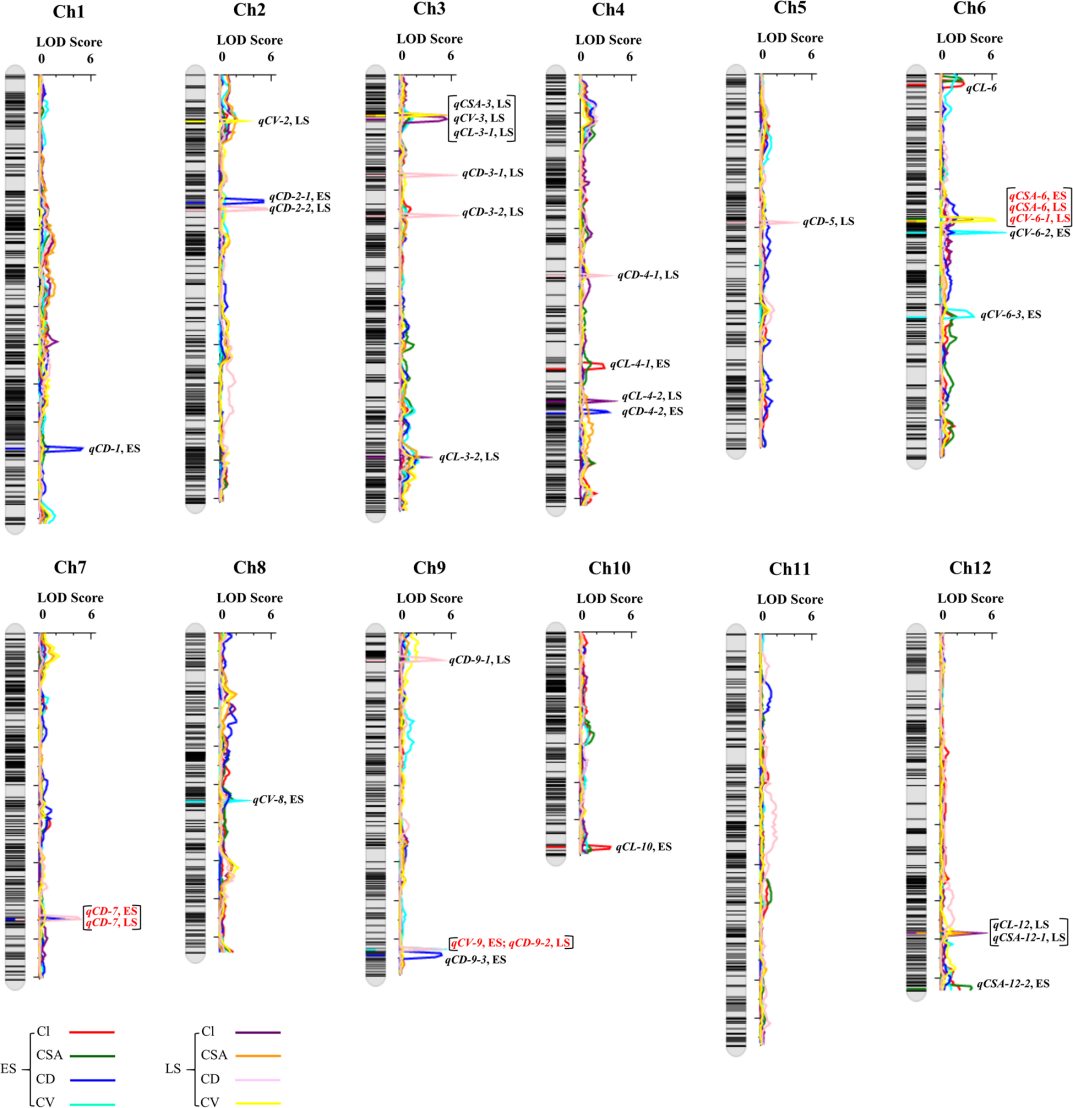
The QTL position on the high-density bin map of the YZX × 02428 RIL population. Square brackets indicate that different QTLs are located at the same physical location on the chromosome. The red text indicates the QTLs that are mapped in the two cropping seasons

The genetic linkage map had an average distance of 0.86 cM between adjacent bin markers and an average physical distance between the markers of 137.68 Kb. Using the inclusive composite interval mapping (ICIM) method, a total of 32 QTLs affecting CL, CSA, CV, and CD were detected across the two cropping seasons and were distributed on all chromosomes, except chromosome 11. In all QTLs, some showed a negative additive effect and the other showed a positive additive effect, indicating that both parents contributed favorable alleles. Seven QTLs were associated with CL and each accounted for 4.99%~ 10.30% of the phenotypic variation. Five QTLs were identified for CSA and each accounted for 5.93%~ 10.35% of the phenotypic variation. Seven QTLs were related to CV and each contributed 5.19%~ 12.83% of the phenotypic variation. Thirteen QTLs were associated with CD and each explained 4.28%~ 9.81% of the phenotypic variation. The QTLs that overlapped based on physical position were classified as the same loci. Ultimately, a total of 25 loci were obtained. Notably, one of the 25 loci were repeatedly detected across the two seasons, that is, *qCD-7* (ES) and *qCD-7* (LS). Two loci were detected by different traits, that is, *qCSA-3*, *qCV-3* and *qCL-3-1*; *qCL-12* and *qCSA-12–1*, and they were detected at LS. In addition, two loci were detected by different traits across the two seasons, that is, *qCSA-6* (ES), *qCSA-6* (LS), and *qCV-6-1* (LS); *qCV-9* (ES) and *qCD-9-2* (LS) (Table 2 and Fig. 2).

**Table 2:** QTLs associated with coleoptile phenotypes under hypoxic conditions detected in the two cropping seasons

QTL	Environment^a^	Chr.^b^	Marker interval	Physical interval (bp)	LOD^c^	PVE(%)^d^	ADD^e^	known loci
*qCD-1*	ES	1	mk258-mk259	38,550,000-38,900,000	5.15	9.73	0.0148	Hsu and Tung, 2015
*qCV-2*	LS	2	mk312-mk313	1,650,000-1,750,000	2.80	5.19	−0.0004	
*qCD-2-1*	ES	2	mk368-mk369	8,950,000-9,150,000	4.95	9.26	−0.0141	
*qCD-2-2*	LS	2	mk375-mk376	9,850,000-9,950,000	5.54	8.72	−0.0138	*qSHL2.2*, Manangkil et al., 2013
*qCSA-3*	LS	3	mk589-mk593	4,750,000-5,150,000	4.40	10.35	0.0315	
*qCV-3*	LS				3.44	6.53	0.0005	
*qCL-3-1*	LS				5.35	10.30	0.1586	
*qCD-3-1*	LS	3	mk623-mk624	8,350,000-8,450,000	5.63	9.09	−0.0138	*qSAT-3-B*, Wang et al., 2009
*qCD-3-2*	LS	3	mk646-mk647	10,950,000–11,050,000	5.84	9.81	0.0166	Hsu and Tung, 2015
*qCL-3-2*	LS	3	mk818-mk819	31,050,000-31,150,000	2.67	4.99	0.1066	
*qCD-4-1*	LS	4	mk1005-mk1006	19,450,000–19,550,000	2.85	4.28	0.0095	*qSHL4.1*, Manangkil et al., 2013
*qCL-4-1*	ES	4	mk1033-mk1034	22,250,000-22,350,000	2.81	6.04	−0.1308	
*qCL-4-2*	LS	4	mk1047-mk1052	23,700,000-24,450,000	3.30	6.44	−0.1224	
*qCD-4-2*	ES	4	mk1063-mk1064	25,800,000-26,100,000	3.52	6.46	0.0116	
*qCD-5*	LS	5	mk1219-mk1220	15,850,000–15,950,000	3.52	5.32	0.0106	
*qCL-6*	ES	6	mk1329-mk1330	550,000-650,000	2.69	6.02	0.1304	Zhang et al., 2017
*qCSA-6*	ES	6	mk1402-mk1406	9,850,000–10,300,000	3.67	8.65	0.0338	Zhang et al., 2017
*qCSA-6*	LS				3.43	8.19	0.0268	
*qCV-6-1*	LS				6.27	12.83	0.0006	
*qCV-6-2*	ES	6	mk1426-mk1427	13,450,000–13,700,000	6.56	12.65	0.0008	
*qCV-6-3*	ES	6	mk1489-mk1490	22,250,000-22,350,000	3.82	7.30	−0.0006	
*qCD-7*	ES	7	mk1758-mk1759	26,550,000-26,650,000	2.86	5.16	0.0107	*qAG7–2*, Angaji et al., 2010
*qCD-7*	LS				4.67	7.13	0.0124	
*qCV-8*	ES	8	mk1886-mk1887	17,300,000–17,500,000	2.60	4.84	0.0005	
*qCD-9-1*	LS	9	mk1990-mk1991	4,450,000-4,600,000	4.70	7.19	0.0125	
*qCV-9*	ES	9	mk2124-mk2125	20,850,000-20,950,000	4.37	8.28	0.0007	
*qCD-9-2*	LS				4.05	6.33	0.0114	
*qCD-9-3*	ES	9	mk2130-mk2131	21,450,000-21,600,000	5.08	9.65	0.0144	
*qCL-10*	ES	10	mk2299-mk2300	22,350,000-22,550,000	3.44	7.41	0.1445	Hsu and Tung, 2015
*qCL-12*	LS	12	mk2668-mk2673	21,250,000-21,900,000	4.35	8.29	−0.1375	
*qCSA-12–1*	LS				2.66	5.93	−0.0227	
*qCSA-12–2*	ES	12	mk2708-mk2709	26,800,000-26,950,000	3.57	8.57	−0.0339	Hsu and Tung, 2015

^a^Environment: ES is early cropping season in 2016; LS is late cropping season in 2016

^b^Chr., chromosome

^c^LOD, logarithm of odds

^d^PVE (%), phenotypic variation explained (%)

^e^ADD, additive effect; a positive value indicates the superiority of *japonica* 02428

We discovered that up to 10 of the 25 loci have been reported previously (Table 2). *qCD-1, qCD-3-2, qCL-10,* and *qCSA-12–2* overlapped with four loci associated with the anaerobic response index [26]. *qCD-2-2* and *qCD-4-1* were located within the genomic region of *qSHL2.2* and *qSHL4.1,* respectively [21]. *qCD-3-1* was mapped to the genomic region of *qSAT-3-B* [19]. The physical position of *qCD-7* was in accordance with the genomic region of *qAG7–2,* which controlled survival rate under anaerobic conditions [4]. The loci consisting of *qCSA-6* and *qCV-6-1* (Chr.:9,850,000–10,300,000) overlapped with the loci associated with coleoptile length at room temperature, *qCL-6* overlapped with the loci associated with flooding tolerance index [27]. The above reported results indicate the accuracy of our mapping results.

### Effects analysis of the stable QTLs

Three types of QTLs were defined as stable QTLs, including overlapped QTLs associated with multiple traits in a single environment; QTLs repeatedly detected in the two environments; and the QTLs that could be co-located with previous reports. In this study, a total of 13 loci consisting of 20 stable QTLs were obtained (Table 3).

**Table 3:** Information on the 20 stable QTLs

Loci	QTL	Marker interval	Physical interval (bp)
loci1	*qCD-1*(ES)	mk258-mk259	Chr.1: 38,550,000-38,900,000
loci2	*qCD-2-2*(LS)	mk375-mk376	Chr.2: 9,850,000-9,950,000
loci3	*qCL-3-1*(LS)*; qCSA-3*(LS); *qCV-3*(LS)	mk589-mk593	Chr.3: 4,750,000-5,150,000
loci4	*qCD-3-1*(LS)	mk623-mk624	Chr.3: 8,350,000-8,450,000
loci5	*qCD-3-2*(LS)	mk646-mk647	Chr.3: 10,950,000–11,050,000
loci6	*qCD-4-1*(LS)	mk1005-mk1006	Chr.4: 19,450,000–19,550,000
loci7	*qCL-6*(ES)	mk1329-mk1330	Chr.6: 550,000-650,000
loci8	*qCV-6-1*(LS); *qCSA-6*(ES); *qCSA-6*(LS)	mk1402-mk1406	Chr.6: 9,850,000–10,300,000
loci9	*qCD-7*(ES); *qCD-7*(LS)	mk1758-mk1759	Chr.7: 26,550,000-26,650,000
loci10	*qCV-9*(ES); *qCD-9-2*(LS)	mk2124-mk2125	Chr.9: 20,850,000-20,950,000
loci11	*qCL-10*(ES)	mk2299-mk2300	Chr.10: 22,350,000-22,550,000
loci12	*qCSA-12–1*(LS);*qCL-12*(LS)	mk2668-mk2673	Chr.12: 21,250,000-21,900,000
loci13	*qCSA-12–2*(ES)	mk2708-mk2709	Chr.12: 26,800,000-26,950,000

To further clarify the effects of the stable QTLs, we summarized the phenotypic differences between the two alleles of each locus in the RIL population (Additional file 3: Table S3). We first divided the individuals of the RIL population into 02428 types and YZX types according to the marker genotypes of each identified locus and then compared the differences between the two genotypes for the corresponding traits.

Among the 13 loci, eight loci showed stable and reliable effects for all four traits. For these eight loci, the mean value of the corresponding traits of the individuals with elite alleles was greater than the individuals with nonelite alleles in the RIL population, and some even reached statistical significance. This indicated that these eight loci, although partially affected by the environment, were reliable and responsible for the phenotypic variation. The other five loci were associated with CD or CV, and the mean value of the individuals that harbored elite alleles was superior to the mean value of the nonelite alleles. For the effect of a single locus, using the absolute value of the difference between two alleles as the standard, loci3 consisting of *qCL-3-1* (LS), *qCSA-3* (LS), and *qCV-3* (LS) had the largest absolute value of CL (0.30 cm) at LS. Loci8 consisting of *qCV-6-1* (LS), *qCSA-6* (ES), and *qCSA-6* (LS) had the largest absolute value of CSA (0.07 cm^2^) at ES. Loci10 consisting of *qCV-9* (ES) and *qCD-9-2* (LS) had the largest absolute value of CV (1.65 mm^3^) and CD (0.04 mm) at ES and LS, respectively. These results suggested that all these loci could be efficiently used for molecular breeding.

**Fig. 3:**
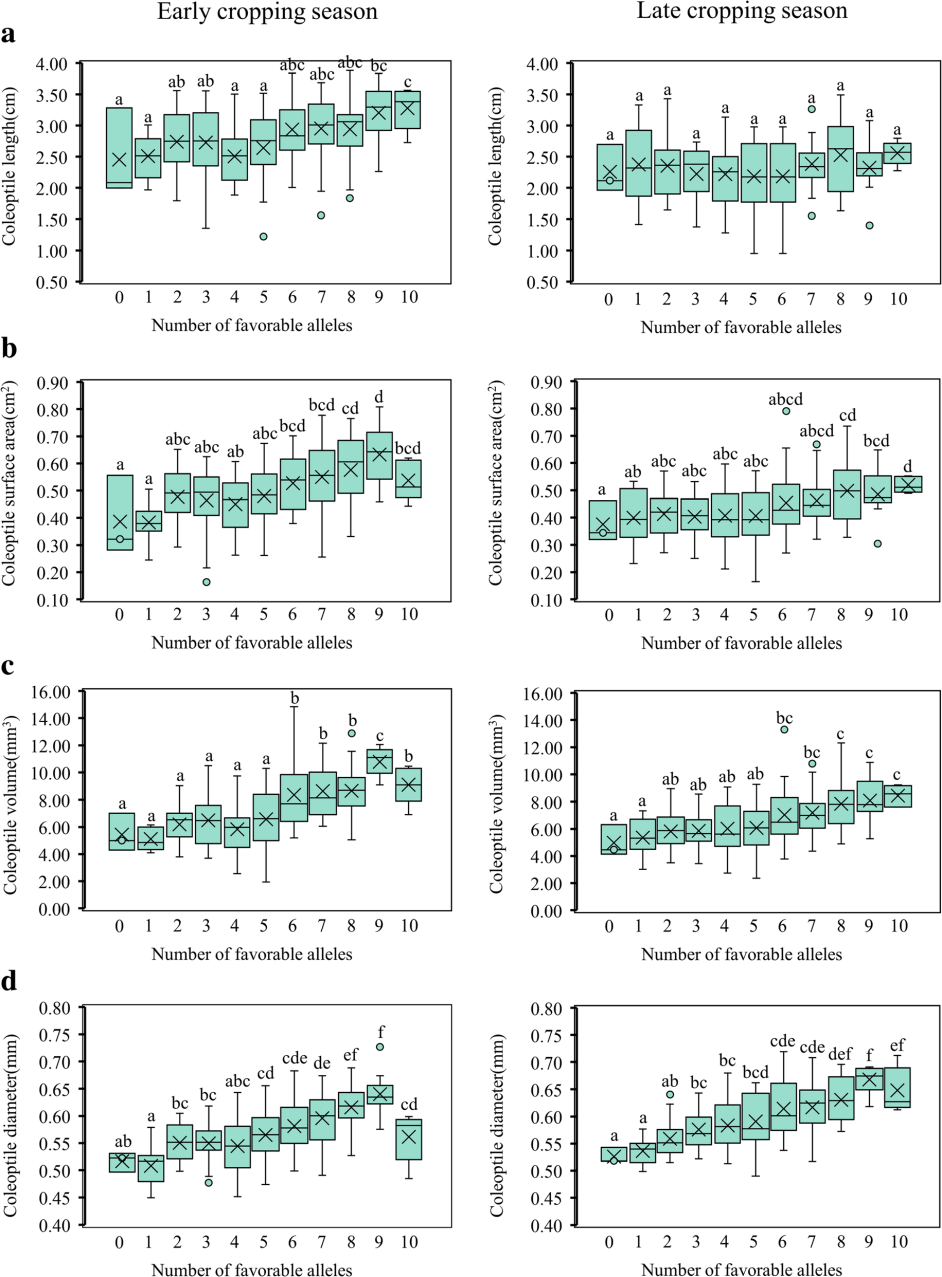
Pyramiding of favorable alleles. **a** – **d** represent CL, CSA, CV, and CD, respectively. Letters from **a** to **f** indicate significantly different values according to statistical analysis using Duncan’s Multiple Range Test (alpha = 0.05)

To confirm this, the elite alleles were used to test the effectiveness of pyramid breeding. Without considering the interactive effects among these 13 stable loci and environmental influences, the more elite alleles that were pyramided in an individual, the higher the values of these traits were in the two cropping seasons (Fig. 3). This result indicated that the pyramiding of favorable alleles could improve AG tolerance. Practically, AG tolerance could be modified via a combination of different numbers of favorable alleles in the varieties. In the RIL population, most individuals harbored four to seven favorable alleles, few harbored zero favorable alleles, and some individuals harbored up to 10 favorable alleles. Therefore, we also obtained accessions (G9, G10, G16, and G151) that carried more than eight elite alleles and exhibited superior AG tolerance ability (Additional file 4: Table S4) and could serve as favorable alleles donor parents in the breeding procedure.

### Identification of candidate genes within the 13 stable loci via gene expression profiling

**Fig. 4:**
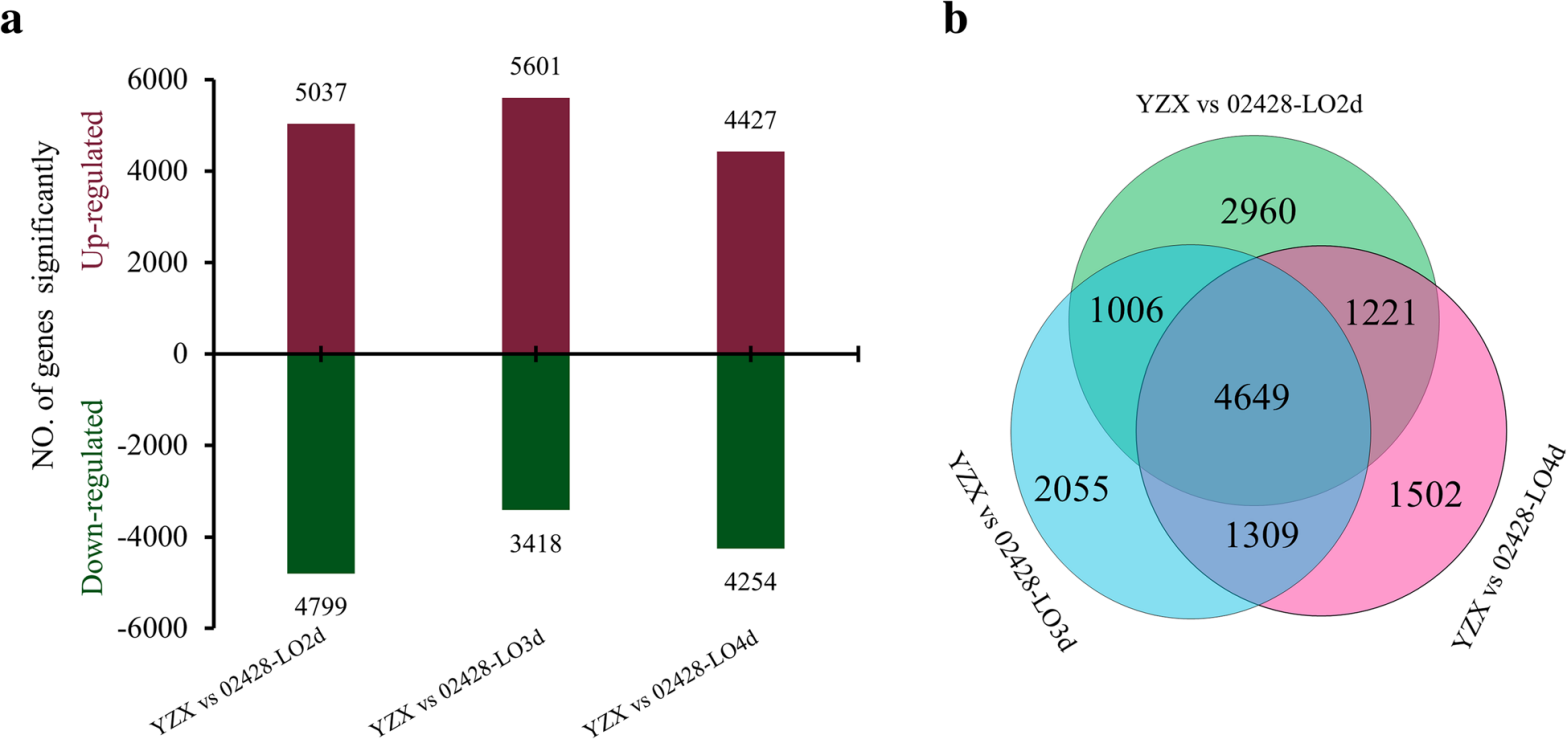
DEGs between the parents on different days of germination. **a** Up and down regulated genes detected between the two parents on different days of germination. **b** Venn diagram of DEGs between the parents on different days of germination

Considering that coleoptile growth in the two parents occurred mainly from the 2nd to the 5th day, and the differences between the two parents were significant, RNA-Seq analysis was performed on the whole germinating seeds, including the embryo and coleoptile, on days two, three, and four under anaerobic conditions. Through differential expression profiling analysis (FDR ≤ 0.05, absolute value fold change ≥ 1.5) of the two parents at different sampling points, the DEGs were identified for the corresponding days. A total of 10,053 DEGs were identified during the three periods (Fig. 4).

Such a large number of DEGs could not be responsible for the variation in anaerobic germination ability between YZX and 02428. Thus, functional annotations of the DEGs (YZX-LO versus 02428-LO) on the 2nd, 3rd, and 4th day were analyzed and GO enrichment was performed. We screened all GO terms with *P* < 0.05 (Additional file 5: Figure S1). Notably, we found that “response to stress,” “response to abiotic stimulus,” and “response to stimulus” were highly significantly enriched for all three sampling points. Another study conducted a large-scale transcriptomic analysis of the developing coleoptile and mature coleoptile under different durations of anaerobic conditions [29]. In their study, GO analysis of up-regulated and down-regulated DEGs (aerobic vs. anaerobic) indicated that the “response to stress” and “response to stimulus” terms were significantly enriched, which corroborates the present study.

Of the 13 stable loci, three were newly identified in this study and 10 overlapped with previous reports whose candidate genes responsible for traits were unknown. In order to further explain the mechanism of AG tolerance, we combined the expression profile data to analyze the genes located in these 13 stable loci intervals. A total of 84 candidate DEGs were obtained (Additional file 6: Table S6). The *qCD-4-1* (LS) locus contains only one gene, while the *qCL-3-1* (LS), *qCSA-3* (LS), and *qCV-3* (LS) loci contain 20 genes.

**Fig. 5:**
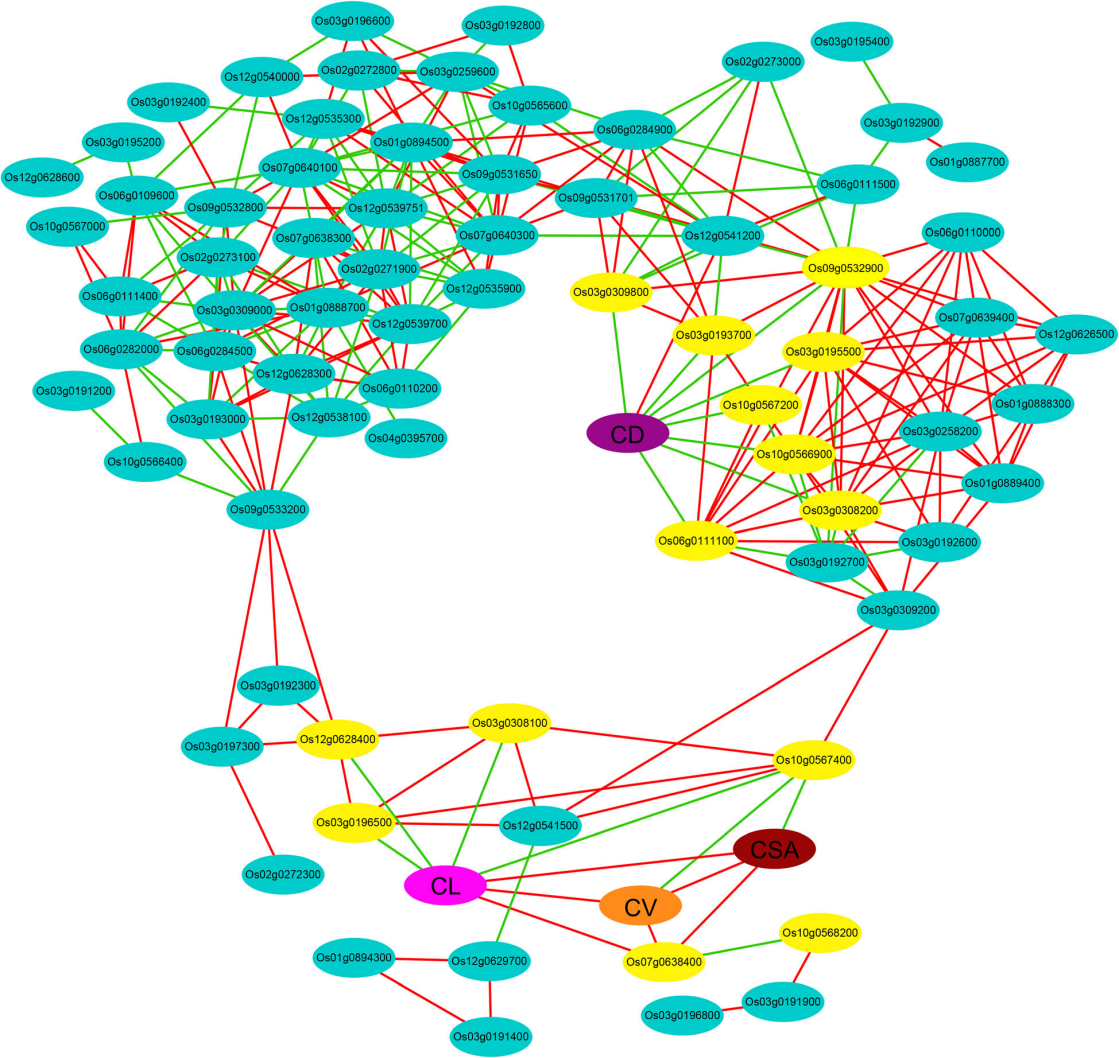
Visualization analysis based on Pearson correlations between genes and the phenotype of CL, CSA, CV and CD. Nodes are genes and traits (CL, CSA, CV and CD); edges are their correlation coefficient values, red and green indicate positive and negative, respectively. The yellow node indicates a strong correlation between the gene and the trait

To further narrow down the scope of candidate genes, the fragments per kilo base of exon per million fragments mapped value of 84 genes and the phenotypic value of the four traits (CL, CSA, CV and CD) at three phases were used to calculate the Pearson correlation coefficients in R software. Taking *p* value<0.05 and the absolute value of correlation coefficient>0.9 as the threshold; based on the method [30], the correlation network was constructed with genes and traits as nodes and correlation coefficients as edge. As shown in Fig. 5, the visualized network was divided into three distinct subnetworks. Genes associated with CL, CSA and CV mainly fell into one cluster, mainly including six highly correlated genes. Another cluster consisted of genes associated with CD, mainly including eight highly correlated genes. The 3rd subnetwork is composed of the remaining genes.

**Fig. 6:**
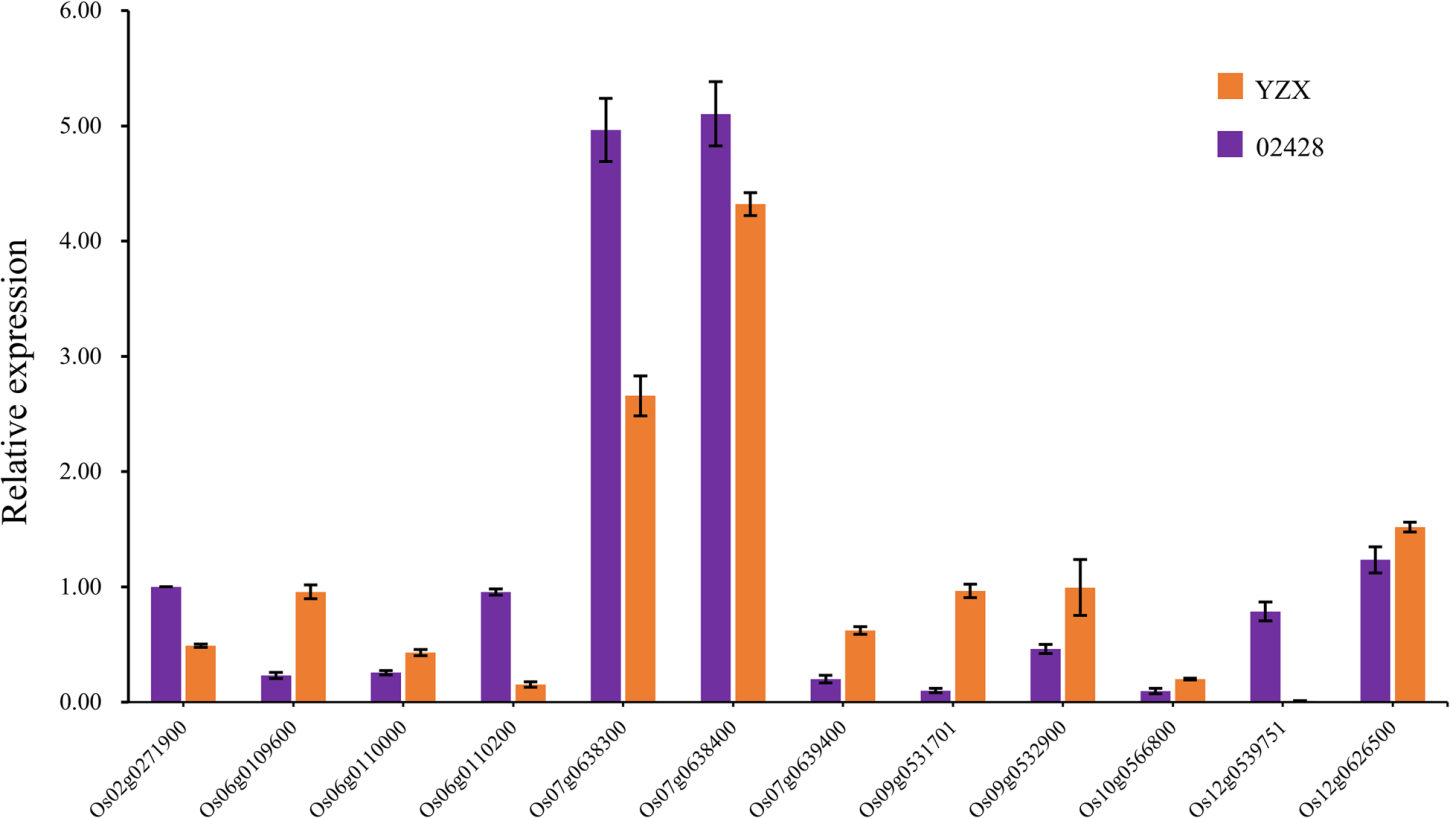
Expression levels of 12 highly promising candidate genes detected by qRT-PCR

Based on the gene annotation (http://rice.plantbiology.msu.edu/), gene differential expression multiples between two parents and correlation with traits. Twelve highly promising candidate genes were identified, including Os02g0271900, Os06g0109600, Os06g0110000, Os06g0110200, Os07g0638300, Os07g0638400, Os07g0639400, Os09g0531701, Os09g0532900, Os10g0566800, Os12g0539751 and Os12g0626500 (Table 4). Notably, the expression levels of Os07g0638400 and Os09g0532900 differ greatly between parents, and they are also highly correlated with traits. We further validated the expression levels of these 12 genes using qRT-PCR in YZX and 02428 seeds after 4 days anaerobic treatment. Which gave similar results to those of RNA-seq analysis – suggesting that our results were reliable (Fig. 6).

**Table 4:** The 12 most promising candidate genes identified from the stable loci

QTL	Gene ID	Fold change(YZX / 02428)			Description
		2d	3d	4d	
*qCAD-2-2*	Os02g0271900	−2.38	−1.90	−2.34	MYB family transcription factor, putative, expressed.
*qCL-6*	Os06g0109600	4.21	5.42	11.14	Adenylate kinase, putative, expressed.
	Os06g0110000	4.82	1.47	2.12	Cytochrome P450, putative, expressed.
	Os06g0110200	− 100.58	−730.33	−7.24	Late embryogenesis abundant group 1, putative, expressed.
*qCD-7*(ES)*; qCD-7*(LS)	Os07g0638300	−10.94	−4.92	−2.98	Peroxiredoxin, putative, expressed.
	Os07g0638400	−2.97	1.27	−1.04	Peroxiredoxin, putative, expressed.
	Os07g0639400	5.71	1.73	2.87	Peroxidase precursor, putative, expressed.
*qCV-9*(ES)*; qCD-9-2*(LS)	Os09g0531701	129.05	142.11	116.18	Glycosyl transferase family 8 protein, expressed.
	Os09g0532900	2.74	1.99	2.73	MYB family transcription factor, putative, expressed.
*qCL-10*	Os10g0566800	−1.39	−1.03	2.56	Peroxidase precursor, putative, expressed.
*qCL-12; qCSA-12–1*	Os12g0539751	−450.33	− 441.33	− 377.33	Expressed protein.
*qCSA-12–2*	Os12g0626500	3.33	1.61	1.19	Late embryogenesis abundant protein D-34, putative, expressed.

## Discussion

### Additional AG tolerance indicators should be developed for the evaluation of AG tolerance

To date, two main indicators have been used to identify the AG tolerance capacity of rice seeds. One indicator is the seedling survival ratio after 21 days of submergence under 10 or 20 cm water head, which is a standardized method developed by International Rice Research Institute [4, 23]. Using this method, some germplasms with good AG tolerance have been screened, and several QTLs were identified [4, 23–25]. Notably, *OsTPP7* in *qAG-9-2* has been cloned and functionally validated [13], indicating that this method is feasible. However, the survival ratio method is labor- and time-intensive and laborious. One alternative method is to use a phenotype associated with coleoptile elongation as an indicator. The QTLs obtained using coleoptile elongation can be overlapped with those obtained using the survival rate [26, 27]. In this study, coleoptile traits were validated using QTL mapping (Table 2). However, only seven QTLs were identified for CL, while 25 QTLs were identified for CV, CSA, and CD. Our QTL mapping results indicate that the use of CL as the only indicator is relatively inefficient. Therefore, it is necessary to develop more indicators. Such as days to emergence of first leaf or root, the seedling survival rate that via anaerobic change to aerobic treatment, seedling quality of seedling formed under anaerobic conditions.

### Pyramiding of favorable QTLs can improve AG tolerance in rice

Marker-assisted backcrossing (MABC) has been shown to be an effective strategy. For example, *SUB1* has been introgressed into different global varieties of rice. The swarna-Sub1 and BR11-Sub1 varieties, which were derived from MABC breeding, have shown excellent performance with consistent yield advantages during flash flooding in south Asia [31]. AG tolerance is essential for germination and seedling establishment under anaerobic conditions and is a key trait for DSR varieties. Using the MABC strategy, the QTL containing *OsTPP7* was also transferred into Ciherang-Sub1, which improved the near isogenic lines (NILs) harboring the *SUB1* gene [32]. Phenotypic evaluation showed that the introgression of *AG1* increased AG tolerance compared to Ciherang-Sub1. However, *qAG-9-2* functions well under moderate stress conditions but does not tolerate high stress. Multiple QTLs or genes that combine *qAG-9-2* should enhance AG tolerance.

In this study, 13 stable loci associated with AG tolerance were obtained, and each QTL accounted for 4.84%~ 12.65% of the corresponding phenotypic variation. Moreover, we tested the effectiveness of pyramid breeding using 13 stable loci, and all phenotypes indicated a tendency of increasing phenotypic value with increasing number of favorable loci. Although the RILs carried 10 favorable loci did not exhibit ideal phenotype due to only five RILs harbor 10 favorable loci result in poor representativeness, the RILs contains 7, 8, 9 or more favorable loci have better phenotypic value than that of less than 3 favorable alleles. As a result of transgressive segregation in the RIL population, we obtained some materials that carried multiple favorable alleles and possessed AG tolerance capacity beyond the parents. The identification of stable loci will provide a foundation for the further investigation of the molecular mechanisms of AG tolerance and also provides a material basis for DSR breeding.

### Stable QTLs provide a relevant method for screening promising candidate genes involved in AG tolerance

Generally, increasing marker density is an effective means of improving QTL mapping resolution [33]. In this study, a high-density bin-map for the RIL population was used for QTL mapping in rice for AG tolerance, which significantly increased the QTL mapping resolution compared with traditional markers. However, there were a large number of candidate genes within the identified QTLs intervals. The differential expression profile strategy is a useful approach for studying the genes associated with a given trait [34] and can effectively reduce the number of candidate genes. In our study, we sequenced the transcriptome at three key stages of AG of the two parents, obtained high-quality differential expression profiles, and then mapped the DEGs to 13 target genetic loci and finally obtained only 84 candidate genes, which suggests that the combination of high-density QTL mapping and RNA-Seq constitutes an effective strategy for identifying candidate genes for QTL mapping.

Among the 12 highly promising candidate genes, we found two late embryogenesis abundant (LEA) protein-related genes, Os06g0110200 and Os12g0626500. Notably, Os06g0110200, which was differentially expressed by up to 730-fold between 02428 and YZX, showed extremely low expression in YZX. LEA proteins, which are named for their abundant expression during the later stages of embryo development in plant seeds, are a group of stress-responsive proteins that are induced by environmental stress. The transgenic overexpression of *LEAs* indicated that these proteins play an important role in the tolerance of rice to drought, salt, and abscisic acid [35, 36]. The OsLEA4 protein encoded by Os06g0110200 enhanced the tolerance towards high salinity, heat, freezing, and UV radiation in *E. coli* recombinants [37]. Furthermore, microarray profiling showed that the expression level of Os06g0110200 was sharply reduced after treatment for 3 h under anaerobic conditions [38].

In addition, we found four peroxidase-related genes, of which the expression of Os07g0638300 was increased by up to 10-fold, Os07g0638400 is highly correlated with CL,CSA and CV. Peroxidase activity in germinating seeds incubated under anoxic conditions for 5 d was substantially lower in the tolerant genotypes than in the intolerant genotypes [3]. Transcript profiling of anoxia-grown rice seedlings revealed that a catalase-coding transcript was strongly down-regulated under anoxia [39]. Reactive oxygen species (ROS) are considered as messengers or transmitters of environmental cues during seed germination [40]. Hydrogen peroxide (H_2_O_2_) is a ROS, and the accumulation of H_2_O_2_ has been reported in the early stage of germination imbibition in different plant species [41–43]. The exogenous application of H_2_O_2_ improved seed germination in many plants, including barley, pea, almond, and rice [44–47]. However, the excessive production of ROS by germinating seeds has often been considered as a negative factor that inhibits the germination process. Therefore, antioxidative mechanisms are regarded as being of particular importance for germination success via the conversion of H_2_O_2_ to oxygen and water [48, 49]. Based on known results, we speculated that the large accumulation of H_2_O_2_ promoted seed germination in the early stage of seed germination, while excessive H_2_O_2_ inhibited seed germination in the middle and late stages of seed germination. Thus, the AG tolerance materials exhibited a tendency of shift in gene expression from high to low, while the nonresistant material exhibited the opposite. Therefore, peroxidase can affect the AG ability of rice seeds by regulating the content of H_2_O_2_ in the seeds.

A study reveals two crucial transcription factors, MYBS1 and MYBGA, present in rice (*Oryza sativa*) and barley (*Hordeum vulgare*), that function to integrate diverse nutrient starvation and gibberellin (GA) signaling pathways during germination of cereal grains. Sugar represses but sugar starvation induces MYBS1 synthesis and its nuclear translocation. GA antagonizes sugar repression by enhancing conuclear transport of the GA-inducible MYBGA with MYBS1 and the formation of a stable bipartite MYB-DNA complex to activate the α-amylase gene [50]. In the region containing two QTLs on chromosomes 2 and 9, we identified two genes, namely Os02g0271900 and Os09g0532900, that encode MYB family transcription factors, the functions of which may be similar to those of the homologous genes *OsMYBS1* and *MYBGA* [50].

Os06g0110000 and Os06g0109600, which encode cytochrome P450 and adenylate kinase, respectively, are associated with CL and showed lower expression levels in the tolerant material 02428. The most common reaction catalyzed by cytochrome P450 is a monooxygenase reaction, for example, the insertion of one atom of oxygen into the aliphatic position of an organic substrate (RH), while the other oxygen atom is reduced to water. Os06g0109600 encodes a protein that catalyzes the interconversion of adenine nucleotides (ATP, ADP, and AMP). Microarray profiling shows that the expression level of Os06g0109600 was sharply reduced after treatment for 3 h under anaerobic conditions, while the expression of Os06g0110000 was increased rapidly after 3 h of aerobic treatment [51]. This result indicates that these two genes play a negative role in AG tolerance, as the AG-tolerant materials maintained lower expression levels of these genes. Os09g0531701 encodes a glycosyl transferase family 8 protein that catalyzes the transferal of saccharide moieties from an activated nucleotide sugar (also known as the “glycosyl donor”) to a nucleophilic glycosyl acceptor molecule. The result of glycosyl transfer can be a carbohydrate, glycoside, oligosaccharide, or a polysaccharide. The relationship between the activity of glycosyl transferase and AG tolerance is unknown. In the present study, the expression of Os09g0531701 was significantly reduced by up to 142-fold in the AG-tolerant material 02428, indicating that anaerobic conditions limit the activity of glycosyl transferase. In addition, Os12g0539751 exhibited highly variable expression levels among the parents. All of the above candidate genes provide a basis for the molecular cloning and development of target gene markers.

## Conclusions

In this study, a biparental population with a high-density genetic bin map was used to identify QTLs for rice resistance to AG. CL, CSA, CV, and CD were used as indicators for our phenotypic identification. A total of 25 loci were detected across two cropping seasons. Including the 10 loci that overlapped with previous reports, a total of 13 were defined as stable loci. After verification, pyramiding of the 13 favorable loci could improve AG, which confirmed the accuracy of our mapping results. We sequenced the transcriptome at three key stages of AG of the two parents, obtained high-quality differential expression profiles, mapped the genes into 13 target genetic loci, and ultimately obtained 12 promising candidate genes. These results improve our understanding of the genetic basis of rice seed AG.

## Methods

### Plant material

The mapping population consisted of 192 RILs derived by single-seed descent from an inter-subspecific cross of the *indica* YZX and the *japonica* 02428 [52]. YZX and 02428 were bred by Hunan Academy of Agricultural Sciences and Jiangsu Academy of Agricultural Sciences, respectively. The RILs and parents were grown in a paddy field at the South China Agricultural University, Guangzhou, China (at approximately 113° east longitude and approximately 23° north latitude) at the early cropping season (ES) and late cropping season (LS) in 2016. Each RIL or parent was planted in a block designed of 6 column × 6 row, with spacing of 20 cm among the plants. Crop management, and disease and insect pest control were performed as locally recommended. All the materials come from the germplasm resource bank of the National Engineering Research Center of Plant Space Breeding. Considering that seed maturity affects AG, six individual plants in the middle of each block were harvested independently at the 35th day after heading in ES and the 40th day after heading in LS. The harvested seeds were dried in a heated air dryer at 42 °C for 5 d and then stored at − 20 °C.

### Evaluation of tolerance to anaerobic conditions during germination

From the six independently harvested plants per season, three individuals were selected per line and 20 healthy seeds were selected for each. Seeds were placed in an oven at 50 °C for 7 d to break dormancy, following which they were surface-sterilized with 20% diluted bleach (6–7% NaClO) for 20 min and then thoroughly rinsed with sterile water. Five seeds were placed at bottom of one centrifuge tube (10 cm), which were filled with distilled water to create an oxygen-free environment. All centrifuge tubes were immediately placed in a chamber with an 8 h light (200 μmol m^− 2^ s^− 1^)/16 h dark cycle at 30 °C. After 6 d, a WinRHIZO (Regent Instruments Inc., Québec, Canada) root image analysis system was used to measure the coleoptile length (CL), coleoptile surface area (CSA), coleoptile volume (CV), and coleoptile diameter (CD). Twelve replicates (centrifuge tubes) were performed per line. Statistical analysis was performed with SAS (Statistical Analysis System, version 8.01) and Microsoft Excel.

### QTL analyses

We used 192 RILs derived from the 02428/YZX cross for mapping the QTLs involved in rice seed AG. Using the Genotyping-By-Sequencing approach, 85,742 high-quality SNPs were validated for recombinant event determination. Using a sliding window approach, a high density bin map with an average physical length of 137.68 kb was obtained. Which contained 2711 bin markers that were evenly distributed across 12 chromosomes. The number of markers on each chromosome ranged from 162 to 311. (Chen et al. 2016). QTL IciMapping v4.1 [53] software was used for QTL analyses. The significance threshold value of the logarithm of odds (LOD) scores for QTL detection was 2.5; if the LOD ≥ 2.5, the site was considered to constitute a QTL for the trait, and the additive effect of each QTL and the contribution rate to the trait were calculated.

### RNA sequencing (RNA-Seq) and data analysis

The total RNA of each sample was homogenized using a mortar and pestle with liquid nitrogen and then purified using the Plant Total RNA Purification Kit (ComWin Biotech Company) following the manufacturer’s instructions. RNA-Seq library construction and sequencing followed a previous protocol [52]. Quality control was confirmed within the Illumina HiSeq software and all reads passing the filtering specifications were mapped onto the reference genome IRGSP-1.0.

After the expression level of each transcript and gene was calculated, differential expression analysis was conducted using edgeR [54]. The false discovery rate (FDR) was used to determine the *p-*value threshold in multiple tests, and for the analysis, a threshold of FDR ≤ 0.05 and an absolute value of fold change ≥ 1.5 were used to assess the significance of the gene expression. The differentially expressed genes (DEGs) were used for GO (Gene Ontology) enrichment, and the groups with FDR ≤ 0.05 were considered to be significantly enriched.

The Pearson correlation coefficient was calculated in R software version 3.5.0, and the networks were visualized using Cytoscape version.3.6.1.

### Validation of candidate genes by real-time quantitative RT-PCR

RNA samples were reverse transcribed into cDNA using the high-capacity cDNA Archive kit (Applied Biosystems, USA). qRT-PCR was conducted using the AceQ qPCR SYBR Green Master Mix Kit (Vazyme Biotech) according to standard protocol, and the expression levels of the genes were determined on the StepOnePlus System (Applied Biosystems, USA). Three replicates were taken for each treatment. As an endogenous control, Actin was used for the normalization of Ct value obtained and the relative expression values were calculated by ΔΔCt method. Gene-specific primers were designed using NCBI primer BLAST (http://www.ncbi.nlm.nih.gov/tools/primer-blast/). The primer sequences of the 12 candidate genes are listed in Additional file 7: Table S7.

## Additional files

Additional file 1: **Table S1.** Phenotypic data of all samples. (XLSX 33 kb)

Additional file 2: **Table S2.** Phenotypic correlation coefficients among the CL、CSA、CAD and CV across two cropping seasons in the YZX × 02428 RIL population. * and ** indicate significance levels at *P* < 0.05 and *P* < 0.01, respectively. (XLSX 10 kb)

Additional file 3: **Table S3.** Details of phenotypic difference between two alleles of each loci. ^a,^ phenotype of 0 allele minus that of 2 allele. (XLSX 30 kb)

Additional file 4: **Table S4.** Number of elite alleles contained in each recombinant inbred line. (XLSX 9 kb)

Additional file 5: **Figure S1.** GO over-representation analysis was performed on the DEG sets at different periods. (TIF 2079 kb)

Additional file 6: **Table S5.** Annotated function of differentially expressed genes identified from the stable loci. ^a^ FPKM, fragments per kilo base of exon per million fragments mapped. (XLSX 21 kb)

Additional file 7: **Table S6.** Primers used for qRT-PCR. (XLSX 9 kb)

## Abbreviations

AG: anaerobic germination
CD: coleoptile diameter
CL: coleoptile length
CSA: coleoptile surface area
CV: coleoptile volume
DSR: direct-seeded rice
ES: early cropping season
GWAS: genome-wide sequencing analysis
LS: late cropping season
QTL: quantitative trait loci;

## References

[1] Mahender A, Anandan A, Pradhan SK (2015). Early seedling vigour, an imperative trait for direct-seeded rice: an overview on physio-morphological parameters and molecular markers. Planta.

[2] Yamauchi M, Aguilar AM, Vaughan DA, Seshu DV (1993). Rice (*Oryza sativa* L.) germplasm suitable for direct sowing under flooded soil surface. Euphytica.

[3] Ismail Abdelbagi M., Ella Evangelina S., Vergara Georgina V., Mackill David J. (2008). Mechanisms associated with tolerance to flooding during germination and early seedling growth in rice (Oryza sativa). Annals of Botany.

[4] Angaji S. Abdolhamid, Septiningsih Endang M., Mackill D. J., Ismail Abdelbagi M. (2009). QTLs associated with tolerance of flooding during germination in rice (Oryza sativa L.). Euphytica.

[5] Miro B, Ismail AM. Tolerance of anaerobic conditions caused by flooding during germination and early growth in rice (*Oryza sativa* L.). Front Plant Sci. 2013;4:269. 10.3389/fpls.2013.00269 PMC3719019 23888162

[6] Ismail AM, Johnson DE, Ella ES, Vergara GV, Baltazar AM. Adaptation to flooding during emergence and seedling growth in rice and weeds, and implications for crop establishment. AoB Plants. 2012. 10.1093/aobpla/pls019. 10.1093/aobpla/pls019 PMC3434364 22957137

[7] Perata P, Guglielminetti L, Alpi A (1997). Mobilization of endosperm reserves in cereal seeds under anoxia. Ann Bot.

[8] Hwang YS, Thomas BR, Rodriguez RL (1999). Differential expression of rice alpha-amylase genes during seedling development under anoxia. Plant Mol Biol.

[9] Lee KW, Chen PW, Lu CA, Chen S, Ho THD, Yu SM (2009). Coordinated responses to oxygen and sugar deficiency allow Rice seedlings to tolerate flooding. Sci Signal.

[10] Ricard B (1994). Plant metabolism under hypoxia and anoxia. Plant Physiol Bioch.

[11] Quimio CA, Torrizo LB, Setter TL, Ellis M, Grover A, Abrigo EM, Oliva NP, Ella ES, Carpena AL, Ito O, Peacock WJ, Dennis E, Dattal SK (2000). Enhancement of submergence tolerance in transgenic Rice overproducing pyruvate decarboxylase. J Plant Physiol.

[12] Saika H, Matsumura H, Takano T, Tsutsumi N, Nakazono M (2006). A point mutation of *Adh1* gene is involved in the repression of coleoptile elongation under submergence in Rice. Breeding Sci.

[13] Kretzschmar T, Pelayo MAF, Trijatmiko KR, Gabunada LFM, Alam R, Jimenez R, Mendioro MS, Slamet-Loedin IH, Sreenivasulu N, Bailey-Serres J, Ismail AM, Mackill DJ, Septiningsih EM (2015). A trehalose-6-phosphate phosphatase enhances anaerobic germination tolerance in rice. Nat Plants.

[14] Singh A, Septiningsih EM, Balyan HS, Singh NK, Rai V (2017). Genetics, physiological mechanisms and breeding of flood-tolerant Rice (*Oryza sativa* L.). Plant Cell Physiol.

[15] Magneschi L, Perata P (2009). Rice germination and seedling growth in the absence of oxygen. Ann Bot.

[16] Blokhina O, Fagerstedt KV, Mancuso S, Shabala S (2010). Oxygen deprivation, metabolic adaptations and oxidative stress. Waterlogging Signalling and tolerance in plants.

[17] Jiang L, Hou MY, Wang CM, Wan JM. Quantitative trait loci and epistatic analysis of seed anoxia Germinability in Rice (*Oryza sativa*). Rice Sci. 2004;11:238–44.

[18] Jiang Ling, Liu Shijia, Hou Mingyu, Tang Jiuyou, Chen Liangmin, Zhai Huqu, Wan Jianmin (2006). Analysis of QTLs for seed low temperature germinability and anoxia germinability in rice (Oryza sativa L.). Field Crops Research.

[19] Wang Y. Screening of germplasm adaptable to direct seeding and discovery of favorable alleles for seed vigor and seedling anoxic tolerance in rice (*Oryza sativa* L.). Nanjing: Dissertation; Nanjing Agricultural University; 2009.

[20] Chen S, Wang J, Pan Y, Ma J, Zhang J, Zhang H, Teng S. Genetic analysis of seed germinability under submergence in rice. Chin Bull Bot. 2012;47:28–35.

[21] Manangkil O. E., Vu H. T. T., Mori N., Yoshida S., Nakamura C. (2012). Mapping of quantitative trait loci controlling seedling vigor in rice (Oryza sativa L.) under submergence. Euphytica.

[22] Angaji SA (2008). Mapping QTLs for submergence tolerance during germination in rice. Afa J Biotechnol.

[23] Septiningsih EM, Ignacio JCI, Sendon PMD, Sanchez DL, Ismail AM, Mackill DJ (2013). QTL mapping and confirmation for tolerance of anaerobic conditions during germination derived from the rice landrace Ma-Zhan red. Theor Appl Genet.

[24] Baltazar MD, Ignacio JCI, Thomson MJ, Ismail AM, Mendioro MS, Septiningsih EM (2014). QTL mapping for tolerance of anaerobic germination from IR64 and the aus landrace Nanhi using SNP genotyping. Euphytica.

[25] Kim S, Reinke RF (2018). Identification of QTLs for tolerance to hypoxia during germination in rice. Euphytica.

[26] Hsu S, Tung C (2015). Genetic mapping of anaerobic germination-associated QTLs controlling coleoptile elongation in Rice. Rice.

[27] Zhang M, Lu Q, Wu W, Niu X, Wang C, Feng Y, Xu Q, Wang S, Yuan X, Yu H, Wang Y, Wei X (2017). Association mapping reveals novel genetic loci contributing to flooding tolerance during germination in Indica Rice. Front Plant Sci.

[28] Narsai R, Edwards JM, Roberts TH, Whelan J, Joss GH, Atwell BJ (2015). Mechanisms of growth and patterns of gene expression in oxygen-deprived rice coleoptiles. Plant J.

[29] Narsai R, Secco D, Schultz MD, Ecker JR, Lister R, Whelan J (2017). Dynamic and rapid changes in the transcriptome and epigenome during germination and in developing rice (*Oryza sativa*) coleoptiles under anoxia and re-oxygenation. Plant J.

[30] Smoot ME, Ono K, Ruscheinski J, Wang PL, Ideker T (2010). Cytoscape 2.8: new features for data integration and network visualization. Bioinformatics.

[31] Dar MH, de Janvry A, Emerick K, Raitzer D, Sadoulet E (2013). Flood-tolerant rice reduces yield variability and raises expected yield, differentially benefitting socially disadvantaged groups. Sci Rep.

[32] Toledo AMU, Ignacio JCI, Casal C, Gonzaga ZJ, Mendioro MS, Septiningsih EM (2015). Development of improved Ciherang-Sub1 having tolerance to anaerobic germination conditions. Plant Breed Biotech.

[33] Liu X, Zhang H, Li H, Li N, Zhang Y, Zhang Q, Wang S, Wang Q, Wang H (2008). Fine-mapping quantitative trait loci for body weight and abdominal fat traits: effects of marker density and sample size. Poult Sci.

[34] Lamb J, Crawford ED, Peck D, Modell JW, Blat IC, Wrobel MJ, Lerner J, Brunet J, Subramanian A, Ross KN, Reich M, Hieronymus H, Wei G, Armstrong SA, Haggarty SJ, Clemons PA, Wei R, Carr SA, Lander ES, Golub TR (2006). The connectivity map: using gene-expression signatures to Connect Small Molecules, Genes, and Disease. Science.

[35] Duan J, Cai W (2012). *OsLEA3-2*, an abiotic stress induced gene of Rice plays a key role in salt and drought tolerance. PLoS One.

[36] Xiao B, Huang Y, Tang N, Xiong L (2007). Over-expression of a *LEA* gene in rice improves drought resistance under the field conditions. Theor Appl Genet.

[37] Hu T, Zeng H, He S, Wu Y, Wang G, Huang X (2012). Molecular analysis of OsLEA4 and its contributions to Improve *E. coli* viability. Appl Biochem Biotech.

[38] Narsai R, Howell KA, Carroll A, Ivanova A, Millar AH, Whelan J (2009). Defining core metabolic and transcriptomic responses to oxygen availability in rice embryos and young seedlings. Plant Physiol.

[39] Lasanthi-Kudahettige R, Magneschi L, Loreti E, Gonzali S, Licausi F, Novi G, Beretta O, Vitulli F, Alpi A, Perata P (2007). Transcript profiling of the anoxic Rice coleoptile. Plant Physiol.

[40] Bailly C, El-Maarouf-Bouteau H, Corbineau F (2008). From intracellular signaling networks to cell death: the dual role of reactive oxygen species in seed physiology. CR Biol.

[41] Puntarulo S, Galleano M, Sanchez RA (1991). Boveris a (1991) superoxide anion and hydrogen peroxide metabolism in soybean embryonic axes during germination. BBA-Gen Subjects.

[42] Schopfer P (2001). Hydroxyl radical-induced cell-wall loosening in vitro and in vivo: implications for the control of elongation growth. Plant J.

[43] Bailly C (2004). Active oxygen species and antioxidants in seed biology. Seed Sci Res.

[44] Fontaine O, Huault C, Pavis N, Billard JP (1994). Dormancy breakage of *Hordeum vulgare* seeds: effect of hydrogen peroxide and stratification on glutathione level and glutathione reductase activity. Plant Physiol Bioch.

[45] Barba-Espin G, Diaz-Vivancos P, Clemente-Moreno MJ, Albacete A, Faize L, Faize M, Pérez-Alfocea F, Hernández JA (2010). Interaction between hydrogen peroxide and plant hormones during germination and the early growth of pea seedlings. Plant Cell Environ.

[46] Zeinalabedini M, Majourhat K, Khayamnekoui M, Hernández JA, Martínezgómez P (2009). Breaking seed dormancy in long-term stored seeds from Iranian wild almond species. Seed Sci Technol.

[47] Zhang D, Chen L, Li D, Lv B, Chen Y, Chen J, XuejiaoYan LJ (2014). *OsRACK1* is involved in abscisic acid- and H_2_O_2_-mediated signaling to regulate seed germination in Rice (*Oryza sativa,* L.). PLoS One.

[48] De Gara L, De Pinto MC, Arrigoni O (1997). Ascorbate synthesis and ascorbate peroxidase activity during the early stage of wheat germination. Physiol Plantarum.

[49] Tommasi F, Paciolla C, de Pinto MC, De Gara L (2001). A comparative study of glutathione and ascorbate metabolism during germination of *Pinus pinea* L. seeds. J Exp Bot.

[50] Hong YF, Ho THD, Wu CF, Ho SL, Yeh RH, Lu CA, Chen PW, Yu LC, Chao A, Yu SM (2012). Convergent starvation signals and hormone crosstalk in regulating nutrient mobilization upon germination in cereals. Plant Cell.

[51] Howell KA, Narsai R, Carroll A, Ivanova A, Lohse M, Usadel B, Millar AH (2008). Whelan J (2008) mapping metabolic and transcript temporal switches during germination in Rice highlights specific transcription factors and the role of RNA instability in the germination process. Plant Physiol.

[52] Chen L, Gao W, Chen S, Wang L, Zou J, Liu Y, Wang H, Chen Z, Guo T (2016). High-resolution QTL mapping for grain appearance traits and co-localization of chalkiness-associated differentially expressed candidate genes in rice. Rice.

[53] Meng L, Li H, Zhang L, Wang J (2015). QTL IciMapping: integrated software for genetic linkage map construction and quantitative trait locus mapping in biparental populations. Crop J.

[54] Robinson MD, McCarthy DJ, Smyth GK (2009). edgeR: a Bioconductor package for differential expression analysis of digital gene expression data. Bioinformatics.

