# Supplementary figures and images for: Identification of stable QTLs and candidate genes involved in anaerobic germination tolerance in rice via high-density genetic mapping and RNA-Seq
Source: BMC Genomics. 2019 May 9;20:355. doi: 10.1186/s12864-019-5741-y (PMC6506967; doi:10.1186/s12864-019-5741-y)

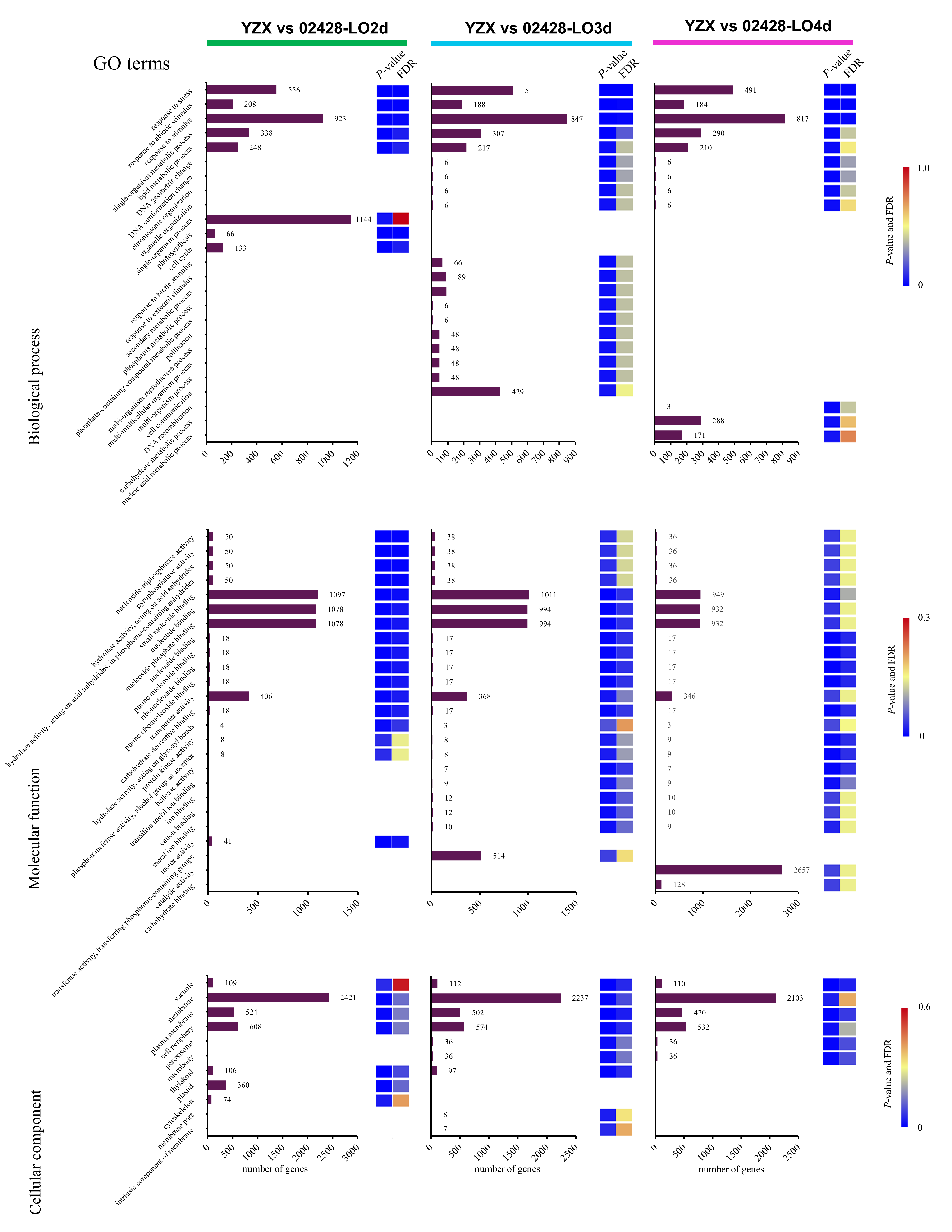

Supplement: Supplementary file 5 — Figure S1. GO over-representation analysis was performed on the DEG sets at different periods. (TIF 2079 kb) [file 12864_2019_5741_MOESM5_ESM.tif]
